# Risk factors associated with vestibulocochlear nerve schwannoma: systematic review

**DOI:** 10.1016/S1808-8694(15)30501-2

**Published:** 2015-10-19

**Authors:** Ana Paula Corona, Jacqueline Carneiro Oliveira, Fàbia Pinheiro Andrade de Souza, Liane Viana Santana, Marco Antônio Vasconcelos Rêgo

**Affiliations:** 1 Speech and Hearing Therapy Department – Federal University of Bahia; 2 Undergraduation, Clinical speech and hearing therapist; 3 Undergraduation, Clinical speech and hearing therapist; 4 Undergraduation, Clinical speech and hearing therapist; 5 Adjunct Professor – Department of Preventive and Social Medicine – Medical School - Federal University of Bahia. Universidade Federal da Bahia

**Keywords:** risk factors, vestibulocochlear nerve, schwannoma

## Abstract

The vestibulocochlear nerve schwannoma (VS) is a benign tumor that stems from the edge of the Schwann's sheath. It is considered the most frequent intracranial benign tumor, of low lethality rate and unknown etiology.

**Aim:**

to identify risk factors associated with VS.

**Study design:**

systematic review.

**Methods:**

electronic search of studies using the following key words: “risk”, “schwannoma”, “vestibular”, “neuroma” and “acoustic”. All original articles on epidemiological studies published in Portuguese, English or Spanish describing measures of association were included.

**Results:**

twenty case-control studies were found, most of them published in the United States. The analysis of those studies shows educational level, household income, occupation, exposure to ionizing radiation and noise, allergic diseases as well as the use of both cellular and cordless phones as risk factors for the VS.

**Conclusion:**

methodological limitations and lack of precision in the findings impose limits to definitive conclusions concerning those risk factors. The current study contributes with information which can subsidize decisions related to the methodology to be used, having in mind new investigations on risk factors for VS. Therefore, it is of great help for knowledge improvement in this field.

## INTRODUCTION

The vestibular nerve schwannoma (VS) is a benign tumor that stems from the Schwann's sheath of one of the vestibular nerves. It is considered the most frequent among intracranial benign tumors - representing 90% of the ponto-cerebelar angle tumors and from 8 to 10% of all the cranial tumors. It is unilateral in about 95% of the cases. Bilateral cases are usually associated with type II neurofibromatosis^1^, ^2^, ^3^, ^4^, ^5^. In most of the cases, this tumor starts between 20 and 60 years of age. It is not associated to any specific race^1^,^4^,^5^ and it is more common in women at a 3:2 ratio. However, its unilateral manifestation seems to be more common in men^6^.

The world incidence rate varies from one to 20 for every 1,000,000 inhabitants per year. In the United States^1^,^3^ and Demark^7^ the estimate is of ten cases per 1,000,000 inhabitants/year. In Brazil there are no estimates of its incidence. Nonetheless, considering these statistics, we believe there are 1,700 new cases per year to be diagnosed. Nonetheless, this figure could be larger, because these numbers are related only to symptomatic cases^4^.

Although it is not a malignant tumor and bears low lethality, suspicion and diagnostic investigation happen only when the first signs and symptoms appear. Nonetheless, even with early diagnosis, the surgical excision of the VS can leave sequelae, such as profound hearing loss and facial paralysis^1^, ^3^, ^4^.

The VS etiology is still obscure. Apparently, the only cause established is a defect on the NF-2 gene of the chromosome 22 long arm, responsible for producing the schwannonian protein, which regulates Schwann cell division4. Such defect can be seen in patients with type II neurofibromatosis; however it is still not proven in patients with unilateral VS.

Currently, different factors associated with brain tumors have been investigated as possible causes for VS. Studies aimed at identifying factors associated to VS development represent a recent task and there are no reports of investigations carried out in Brazil. Thus, the goal of this systematic review is to discuss the main epidemiological findings which analyze the association between the many risk factors and VS.

## MATERIALS AND METHODS

This is a systematic review study on the risk factors associated with VS. Our study object was the scientific papers present in electronic data banks of indexed journals. To search for the papers we used the following electronic data bases: Cohrane, Latin-American and Caribbean Literature in Health Sciences (LILACS), National Library of Medicine (MEDLINE) and SciELO, through the website: http://www.bireme.br/php/index.php. We used the following keywords combination “risk”, “schwannoma”, “vestibular”, “neuroma” and “acoustic”, in the abstracts to identify the papers. After that stage, all the abstracts located were read and we selected the papers according to the inclusion and exclusion criteria defined for the study.

We included only original epidemiology research papers published in Portuguese, Spanish or English, from 1966 to November of 2006, which investigated risk factors associated with VS, and which reported some measure of association. All the literature review papers, case reports or even letters to the editor and editorials as well as the papers which described diagnostic procedure, surgical technique and post-op complications were taken off our review. The epidemiological study papers which investigated risk factors for brain tumors and which presented results for VS grouped with another type of tumor were also taken off.

For each one of the papers selected, we carried out a description of the study outline following a guideline including study place, period, design and bases, population, origin, criteria for sample selection, risk factor investigated and exposure check. We also analyzed the methodological aspects in order to discuss those associated with validity, power and biases. For that, we also checked sample size, age range of the participants, participation response rate, use of substitute respondents, study variables and confounding variables control. This assessment was carried out without the help of the models already described in the literature, because after checking the aspects to be evaluated in this study, we observed that the proposals published are more adequate for the analysis of clinical trials. The results from the studies we selected, which investigated the same risk factor for VS, were grouped and thus the minimum and maximum values of the association measures and confidence intervals were presented. For that, when the study variables were stratified in a different way, it was necessary to create new analysis strata. Finally, the results obtained from these investigations were described and analyzed by means of measures of association and confidence intervals.

## RESULTS

We found 265 papers, from which we selected 20. The main reasons to exclude papers were discussions of surgical techniques (34%), literature reviews (13.9%) and case reports (12.7%).

All the studies analyzed were case-control type, 11 of hospital basis and nine of populational basis. Inskip et al.'s study^8^, hospital-based multicentric case-controlled supported the development of nine other studies (Brenner et al.^9^; De Roos et al.^10^; Inskip et al.^11^; Inskip et al.^12^; Rajaraman et al.^13^; Hill et al.^14^; Kleinerman et al.^15^; Rajamaran et al.^16^; De Roos, et al.^17^). Lönn et al.'s study^18^, populational-based case controlled, provided one more investigation regarding risk factors for VS (Edwards et al.^19^) (Table 1, Table 2).

**Table 1:** Methodological characteristics of the studies which analyzed risk factors associated with vestibular nerve schwannoma

Reference	Study period and place	Study population	Risk factor investigated	Exposure check	Confounding variables control	Comments
1 Preston-Martin et al.,1989	USA 1978-1985	Los Angeles male residents	Dental x-ray and occupational noise	Questionnaire deployed to the case/ control pair by one interviewer only	1. Race 2. Year of birth 3. Weekly expo sure to benzene	Objective measure of noise exposure; information related to ionizing radiation exposure (dental x-ray) were validated by comparison with dental records; a reduced number of individuals to analyze head injury and exposure to chemical products; interviewers were not blinded as to the case or control situation.
2 Rodvall et al.,1998	Sweden 1987-1990	Individuals with age between 25 and 74 years, who lived near the Upsala University Hospital	X-rays and dental capping	Questionnaire sent to the participants; dental records	1. Gender 2. Year of birth 3. Parish 4. Dental pros thesis	Small sample; excluding substitute respondents; data from dental x-rays before 25 years were scatter and not analyzed; radiation dose was not estimated; quality of exposure data is questionable.
3 Inskip et al., 2001	USA 1994-1998	Individuals with age equal to or higher than 18 years of age admitted to three reference centers for nervous system tumors (Phoenix, Boston and Pittsburgh) and who understood Spanish and English	Cell phone	Questionnaire personally deployed in the hospitals	1. Hospital 2. Age 3. Gender 4. Race 5. Proximity be tween the house and the hospital	Cases older than controls; the long time of cell phone use was not evaluated; technology of cell phone used was not investigated.
4 Muscat et al., 2002	USA 1997-1999	Individuals with age equal to or higher than 18 years	Cell phone	Questionnaire personally deployed in the hospitals	1. Age 2. Gender 3. Race 4. Hospital 5. Education 6. Occupation 7. Interview mon th and year	This study was carried out one decade after the cell phone was introduced; long time of cell phone use was not evaluated; hearing loss associated with the vestibular nerve schwannoma can impact responses in relation to the side which the cell phone is used.
5 Brenner et al., 2002	USA 1994-1998	Individuals with ages equal to our higher than 18 years, admitted to three reference centers for nervous system tumors (Phoenix, Boston and Pittsburgh) and who understood English or Spanish	Past of allergic or autoimmune disorders	Questionnaire personally deployed in the hospitals	1. Hospital 2. Age 3. Gender 4. Race 5. Proximity between hospital and household	Interviewers were not blind towards the case or control condition; self-reported allergy -except for hay fever; data were not collected in relation to the treatment carried out for the disorders investigated;
6 Hardell et al., 2003	Sweden 1997-2000	Individuals with ages between 20 and 80 years, of both genders, residing in four medical regions in Sweden	Cell phone and wireless phone	Personally deployed questionnaire	1. Gender 2. Age 3. Geographic location	Inclusion of cases with histopathology confirmation; long time of cell phone use was not assessed; interviewers were blind as to the case and control condition.
7 Roos et al., 2003	EUA 1994-1998	Individuals with ages equal to or above 18 years admitted to three reference centers for nervous system tumors (Phoenix, Boston and Pittsburgh) and who understood English or Spanish	Chemical substances	Questionnaire personally deployed in hospitals and blood sample collection for DNA analysis	1. Hospital 2. Age 3. Gender 4. Race 5. Proximity between hospital and household	The variants of the genes evaluated may also be associated with the disorders of the hospital controls.
8 Inskip et al., 2003a	USA 1994-1998	Individuals with age equal to or higher than 18 years admitted to three nervous system tumor reference centers (Phoenix, Boston and Pittsburgh) and who understood English or Spanish	Laterality	Questionnaire personally deployed in the hospitals	1. Education 2. Marital status	The digit-manual laterality can be influenced by socio-cultural factors.
9Inskip et al., 2003b	USA 1994-1998	Individuals of 18 years of age or higher, admitted to three nervous system tumor reference centers (Phoenix, Boston and Pittsburgh) and who understood English or Spanish	Socio-de-mographic metrics	Questionnaire personally deployed in the hospitals	1. Hospital 2. Age 3. Gender 4. Race 5. Proximity between hospital and household	Inclusion of incidental and histologically confirmed cases; low rate of substitute respondents; hospital controls; education and socio-economical status can impact the perception of signs and symptoms and favor access to diagnosis.
10 Chris-tensen et al., 2004	Denmark 2000-2002	Individuals with ages between 20 and 69 years.	Cell phone	Personally deployed questionnaire	1. Age 2. Gender 3. Education 4. Region 5. Marital status 6. Earphones use	Personal interviews; time used to answer the questionnaire was similar for cases and controls; the time of cell phone used was not estimated according to the technology.
11 Rajara-man et al., 2004	USA 1994-1998	Individuals of 18 years of age or higher, admitted to three nervous system tumor reference centers (Phoenix, Boston and Pittsburgh) and who understood English or Spanish	Occupation	Questionnaire personally deployed in hospitals	1. Hospital 2. Aged 3. Gender 4. Race 5. Proximity between hospital and household	Hospital controls; individuals distributed throughout 121 occupational groups; reduced number of individuals by occupational group.
12 Lönn et al., 2004	Sweden 1999-2002	Individuals with ages between 20 and 69 years, residents in three regions within the scope of the Regional Cancer Register (Stockholm, Göteborg and Lund)	Cell phone	Questionnaire personally deployed or by phone or filled out by the very individual	1. Age 2. Gender 3. Residential area 4. Education	Greater rate of participation of cases; a long time of cell phone use was not assessed.
13 Hill et al., 2004	USA 1994-1998	Individuals of 18 years of age or higher, admitted to three nervous system tumor reference centers (Phoenix, Boston and Pittsburgh) and who understood English or Spanish	Family history of cancer	Questionnaire personally deployed in the hospitals	1. Gender 2. Age 3. Race 4. Proximity between hospital and household	Reduced number of observations in the extracts; it was not confirmed whether or not the reported cases of cancer in the family were true; trained interviewers; interview carried out at the most of three weeks after the diagnosis of the cases.
14 Scho-emaker et al., 2005	Great Britain 1999-2004	Individuals residing in the areas of study scope	Cell phone	Questionnaire deployed personally or by telephone	1. Age 2. Gender 3. Region	Possible selection bias because of a higher rate of participation of the cases; hearing loss associated with vestibu-lar nerve schwannoma can influence responses associated with the use of cell phones; individuals who used the cell phone ipsilaterally to the tumor can have an early diagnosis because of a reduced hearing acuity.
15 Hardell et al., 2005	Sweden 2000-2003	Individuals aged between 20 and 80 years, of both genders, residing in the four medical regions of Sweden	Cell phone and wireless phone	Questionnaire deployed personally or by phone	1. Age 2. Gender 3. Socioecono- mic status 4. year of diag nosis	Interviewers were blind to the case and control condition; a greater number of cases with long time of cell phone use in relation to the other studies.
16 Kleiner-man et al., 2005	EUA 1994-1998	Individuals of 18 years of age or higher, admitted to three nervous system tumor reference centers (Phoenix, Boston and Pittsburgh) and who understood English or Spanish	Electromagnetic waves coming from household appliances	Questionnaire deployed personally in hospitals	1. Age 2. Gender 3. Race 4. Proximity between hospital and household	Questionnaire deployed may have not been proper to assess exposure and may have caused a classification error; incomplete questionnaire data; half of the cases and one fourth of the controls needed help to answer the questionnaire; the interruption in device use was not investigated.
17 Edwards et al., 2005	Sweden 1999-2002	Individuals with ages between 20 and 69 years, residents in three regions within the scope of the Regional Cancer Register (Stockholm, Göteborg and Lund)	Occupational and non-occupational noise	Questionnaire made by phone or personally or filled out by the individual him/herself	1. Age 2. Gender 3. Residential area 4. Education 5. Ionizing radiation 6. Cell phone	Greater participation of cases when compared to controls; interviewers not being blind regarding the status of case or control; noise exposure check was not validated by the work/occupation history.
18 Rajama-ran et al., 2005	USA 1994-1998	Individuals of 18 years of age or higher, admitted to three nervous system tumor reference centers (Phoenix, Boston and Pittsburgh) and who understood English or Spanish	Chemicals	Questionnaire personally deployed in the hospitals and blood sample collection for DNA analysis;	1. Hospital 2. Age 3. Gender 4. Race 5. Proximity between hospital and residence	We took off the controls who had disorders that could be associated with the variants of the investigated gene.
19 Forssén et al., 2006	Sweden 1987-1999	Workers residing in Sweden	Occupational exposure to electromagnetic waves	Questionnaire; cense data/ previously built occupational exposure matrix	1. Gender 2. Age	Large sample and good statistical power; randomly selected controls.
20 Roos et al., 2006	USA 1994-1998	Individuals of 18 years of age or higher, admitted to three nervous system tumor reference centers (Phoenix, Boston and Pittsburgh) and who understood English or Spanish	Chemicals	Questionnaire personally deployed in hospitals and blood sample collection for DNA analysis	1. Hospital 2. Age 3. Gender 4. Race 5. Proximity between hospital and household	High rate of participation among cases and controls.

**Table 2:** Characteristics of the cases and controls from the studies which investigated risk factors associated with vestibular nerve schwanno-ma

Reference	Origin and criteria used for selecting the cases	Origin and criteria used to select the controls	Number		Response rate		Substitute respondents	
			CA	CO	CA	CO	CA	CO
1 Preston-Martin et al.,1989	Microscopically confirmed; incidentals; with ages varying between 25 and 69 years at the time of tumor diagnosis; Los Angeles cancer identification program	The closes male neighbor in the sequence of the strings of cases, paired by race and age.	86	86	76%	17 of the initially selected neighbors refused to participate	Not reported	
2 Rodvall et al.,1998	Microscopically confirmed; incidentals	Parish records	36	339	Rate presented was not specific for each tumor group (case=71%; con-trol=82%)		Number presented is not specific for each tumor group (29)	
3 Inskip et al., 2001	Confirmed by RMI or CT scan or microscopically; incidentals.	Individuals admitted to the same hospitals as a variety of non-malignant conditions	96	799	92%	86%	3%	3%
4 Muscat et al., 2002	Microscopically confirmed	Individuals admitted to the same hospitals with a variety of non-malignant conditions	90	86	Not shown		1	–
5 Brenner et al., 2002	Microscopically confirmed or confirmed by image exam; incidentals	Individuals admitted to the same hospitals with a variety of non-malignant conditions and with prior history of autoimmune diseases and allergies	96	799	Rate shown was not specific for each tumor group (case=92%; con-trol=86%)		4%	4%
6 Hardell et al., 2003	Microscopically diagnosed; Regional Cancer Register; incidentals	Sweden population register	51	44	Rate shown was not specific for each tumor group (case=88%; con-trol=91%)		Not reported	
7 Roos et al., 2003	Confirmed by MRI or CT scan, microscopically; incidentals	Individuals admitted to the same hospitals with a variety of non-malignant conditions	79	604	86%	76%	No	
8 Inskip et al., 2003a	Confirmed by MRI or CT scan or microscopically; incidentals	Individuals admitted to the same hospitals with a variety of non-malignant conditions	96	799	Not reported		Not reported	
9 Inskip et al., 2003b	Confirmed by MRI or CT scan or microscopically; incidentals	Individuals admitted to the same hospitals with a variety of non-malignant conditions	96	799	Not reported		3%	3%
10 Christen-sen et al., 2004	Confirmed by MRI or microscopically; incidentals	Central Danish Po-pulational register; randomized	106	212	82%	64%	Not reported	
11 Rajara-man et al., 2004	Confirmed by MRI or CT scan or microscopically; incidentals	Individuals admitted to the same hospitals with a variety of non-malignant conditions	96	799	93%	86%	Not reported	
12 Lönn et al., 2004	Identified by means of collaboration from the neurosur-gery, oncology, neurology and otorhinolaryngology wards from the hospitals in the study area; incidentals	Sweden populational register; randomized	148	604	93%	72%	No	
13 Hill et al., 2004	Confirmed by MRI or CT scan or microscopically; incidentals; did not have a history of central nervous system tumor.	Individuals admitted to the same hospitals with a variety of non-malignant conditions	96	799	98%	86%	1%	3%
14 Scho-emaker et al., 2005	Identified by means of collaboration from the neurosur-gery, oncology, neurology and otorhinolaryngology wards from the hospitals in the study area; incidentals	Individuals never diagnosed with brain cancer; populational register and patient register in clinics; randomized	678	3553	84%	61%	Not reported	
15 Hardell et al., 2005	Histologically confirmed; incidentals	National populational register	84	692	89%	84%	Not reported	
16 Kleiner-man et al., 2005	Confirmed by MRI or CT scan or microscopically; incidentals.	Individuals admitted to the same hospitals with a variety of non-malignant conditions	90	686	79,8%	73,9%	9	42
17 Edwards et al., 2005	Microscopically confirmed or by MRI and CT scan; incidentals	Sweden populational register; randomized	146	564	91%	67%	2	–
18 Rajama-ran et al., 2005	Confirmed by MRI or CT scan or microscopically; incidentals	Individuals admitted to the same hospitals with a variety of non-malignant conditions	67	505	68%	74%	No	
19 Forssén et al., 2006	Sweden cancer register	Sweden populational register; randomized	793	101. 762	100%	100%	No	
20 Roos et al., 2006	Microscopically confirmed or by MRI and CT scan; incidentals	Individuals admitted to the same hospitals with a variety of non-malignant conditions	79	604	98%	86%	No	

MRI: Magnetic resonance image

CA: cases

CO: controls

The first paper included in the present review was published in 1989 and most of the others (75%) were published after 2003. The United States contributed with 12 papers, Sweden with six, Denmark and Great Britain published one each.

Eighteen studies included only incidental cases. Muscat et al.^20^ and Forséen et al.^21^ did not report this information. Most of the studies (80%) described that VS was confirmed histologically or though an image exam. In two papers (Lönn et al.^18^; Schoemaker et al.^22^), there is only the information that the cases were diagnosed or identified through he health care facilities and in one of the reports (Forséen et al.^21^) the cases were recruited with the support from the national cancer register.

In seven populational-base studies, control selection happened through the use of data coming from the populational register and in one of these they also used additional data from medical clinics. In the study led by Rodvall et al.^23^, the parish register was used to select the controls, and in the study carried out by Preston-Martin et al.^24^ the neighbors of the cases participated in the sample. Of all the populational-base studies, only five described that the controls were randomly selected. For all the hospital-base studies we observed that the individuals who made up the control group were submitted to the same hospitals of the cases with a variety of non-malignant conditions.

In two studies, it was noticed that the exposure checking was not restricted to the information provided by the interviewed individuals. Preston-Martin et al.^24^ validated the information obtained in relation to dental x-ray exposure by comparison with dental records. Now, as far as occupational noise is concerned, the same authors only considered exposed those individuals who reported occupations listed in the national survey of occupational risk in the USA (NOHS) as those in occupations which involve exposure to high levels of sound pressure. In the study carried out by Rajamaran et al.^13^, in which the occupation was analyzed as a risk factor for VS, the 121 occupational groups created were based on manuals which classified and coded occupations in the country.

The minimum number of participants was 36 and the maximum was 793 for the cases; and 44 and 101,762 for the controls, respectively. In most of the studies, the mean ages of cases and controls were similar, usually varying between 20 and 70 years of age. The low participation rate among the cases was observed in the study carried out by Rajamarn et al.^16^ (68%) and the study by Schoemaker et al.^22^ which had the lowest response rate among controls (61%). In the remaining studies, this rate varied between 76% to 98% (cases) and between 64% and 86% (controls).

In three studies we noticed a lower participation of the control group individuals, creating a difference of participating individuals above 20% when compared to the cases^18^, ^19^, ^22^ and in three other studies, the participation rate of the VS cases was presented together with that of other types of tumors^9^, ^23^, ^25^. Substitute respondents were interviewed in eight studies^8^, ^9^, ^12^, ^13^, ^14^, ^15^, ^19^, ^23^ and in four of them the participation reached a maximum value of 4%, and similar among case and control groups^8^, ^9^, ^12^, ^14^. All the studies controlled confounding variables, and gender and age variables were tested in all of them.

Table 3, Table 4 show the positive and negative associations among many variables and the VS respectively. Among the variables which revealed a negative association, only one (heated water mattress) was statistically significant.

**Table 3:** Positive associations found in the studies which assessed the risk factors for schwannoma and vestibular nerve.

Risk factor	Positive association measure					Positive association measure Statistically non-significant		
	*OR^a^ OR RR^b^	CI 95%^c^	P value	Reference	*OR or RR	CI 95%	P value	Reference
Family history of cancer								Hill et al., 2004
Stomach					1,6	0,4 – 6,1		
Colon					1,1	0,4 – 3,8		
Prostate					1,6	0,5 – 5,0		
Any malignant tumor					1,2	0,8 – 1,9		
Any malignant tumor in participants ⩾ 50 years					1,4	0,8 – 2,7		
Socio-demographic metrics				Inskip et al., 2003^b^				Inskip et al., 2003b
Education								
≪ 3 years of higher education					1,6	0,9 – 3,1		
≫ 4 years of higher education	3,2 – 3,4	1,5 – 6,7						
Family income								
Self-reported ($1,000) 25 – 74,9					1,7 – 1,8	0,5 – 3,9		
≫ 75 Census data ($1,000)					2,1	0,9 – 4,8		
< 15 – 24,9					1,3	0,3 – 4,6		
25 – 74,9	2,1 – 2,8	1,1 – 6,3						
≫ 75	7,2	2,5 – 20						
Type of health-care insurance Organization (HMO)					1,3	0,7 – 2,4		
Marital status Singles					1,2	0,6 – 2,6		
Religion Jewish					1,9	0,6 – 5,3		
None/others/unknown					1,2 – 2,1	0,3 – 3,8		
Laterality								Inskip et al., 2003a
Left					1,2	0,6 – 2,2		
Dental x-ray (annual)				Preston-Martin et al.,1989				Preston-Martin et al.,1989
Before 25 years of age					2,1	0,87 −5,69	0,11	
After 25 years of age					2,4	0,99 -	0,03	
Before or after 25 years of age	2,3	1,08 −5,12	0,01					
Noise								
Occupational				Preston-Martin et al.,1989; Edwards et al., 2005;	1,43	0,96 −2,13		Preston-Martin et al.,1989; Edwards et al., 2005;
Non-occupational					1,38	0,80 −2,36		
Occupational and/or non-occupational	1,55	1,04 −2,30						
Continuous	1,5 – 1,79	1,11 −2,89						
Impact	2,1	d						
Continuous and/or impact	2,2	d						
Occupational duration /Men				Edwards et al., 2005;			0,02 – 0,11	Edwards et al., 2005
< 5 years					1,71	0,67 −4,38		
5 – 14 years					2,12	0,99 −4,57		
≫ 15 years					1,18	0,60 −2,32		
Exposure duration/Women								
< 5 years					1,24	0,44 −3,52		
5 – 14 years					1,01	0,36 −2,81		
≫ 15 years	3,34	1,32 −8,43	0,024e					
Latency period			0,0029 e					
13 years – 26 years	1,74	1,06 −2,84						
≫ 27 years	2,15	1,19 −3,86						
Occupational (men/dose-response)				Preston-Martin et al.,1989;				Preston-Martin et al.,1989;
< 5 years					2,9	1,00 −8,60		
5 – 14 years					1,7	0,6 – 4,67		
≫ 15 years	3,5	1,12 −11,17	0,02 e					
Allergic disease				Brenner et al., 2002				Brenner et al., 2002
Asthma					1,34	0,73 −2,46		
Hay fever	2,36	1,38 −4,03						
Year of diagnosis							0,51	
< 10 years – 30 years					1,60 – 1,93	0,51 −5,04		
> 30 years	4,37	1,69 −11,28						
Duration			0,16 e					
< 10 years – 30 years	2,93 – 5,43	1,27 −20,40						
> 30 years					1,58	0,74 −3,38		
Insects					1,12	0,49 −2,54		
Feed	3,01	1,06 −8,53						
Other substances	3,81	1,45 −9,99						
Any allergy				1,02	0,64 −1,63			
Autoimmune disease								
Multiple sclerosis				3,60	0,36 −36,21			
Pernicious anemia				1,25	0,26 −6,06			
Electromagnetic waves						Kleinerman et al., 2005		
(electronic home appliances) Hair dryer				1,5	0,7 – 3,3			
Microwaves				1,9	0,2 – 1,6			
Perm brush (women)				1,3	0,6 – 3,1			
Massage machine				1,1	0,6 – 1,9			
Computer (non-occupational use)				1,7	0,9 – 3,2			
Chemical substances								
(gene polymorphism)						Roos et al., 2003; Roos et al., 2006		
GSTP1 105 Val/Val				1,3	0,6 – 2,9			
GSTP1 114 Ala/Val or Val/Val				1,2	0,6 – 2,5			
CYP2E1 R salt				2,3	1,0 – 5,3			
CYP1B1 V432L CG				1,2	0,7 – 2,3			
EPHX1 Y113H CC				1,5	0,6 – 3,6			
GSTM3 A/B				1,1	0,6 – 1,9			
NQO1 P187S CT e TT				1,2 – 1,3	0,2 – 5,5			
Occupational exposure to magnetic fields (50Hz)						Forssén et al., 2006		
0.11 – 0.19 μT				1,02	0,85 −1,24			
0.2 – 0.30 μT				1,05	0,84 −1,31			
≫ 0.30μT				1,08	0,79 −1,47			
Occupation				Rajaraman et al., 2004				Rajaraman et al., 2004
Always worked on this occupation								
Gas station attendants Mechanics and aides	2,4	1,0 – 6,0f			2,0	0,7 – 5,3		
Blacksmiths					2,2	0,8 – 5,9		
Buyers	2,9	1,0 −8,8 f						
Recreation professionals and physical education teachers					1,9	0,7 – 4,9		
Sales rep	1,9	1,0 −3,5 f						
Professors and instructors	1,8	1,0 −3,5 f						
Worked for 5 years in the occupation								
Office worker					1,1	0,6 – 2,1		
Professors and instructors Cell phone					1,7	0,7 – 4,0		
Regular use duration 5 – 9 years of age					1,1	0,7 – 2,0		Lönn et al, 2004
≫ 10 years of age					1,6	0,7 – 3,6		
Cumulative use								
< 5 years					1,03	0,54 −1,95		Christensen et al., 2004
≫ 5 years					1,1	0,4 – 2,8		Schoemaker et al., 2005
Analogue technology								
Regular use					1,6	0,9 – 2,8		Lönn et al.,2004; Schoemaker et al., 2005
Time since the first regular use 5 – 9 years					1,3	0,6 – 2,9		
≫ 10 years Latency					1,1 – 1,8	0,7 – 4,3		
> 1 – 10 years	5,1 – 9,9	1,4 – 69		Hardell et al., 2005				
> 10 years Use side/VS location	1,5	1,02 −2,3		Hardell et al., 2003	2,6	0,9 – 8,0		Hardell et al., 2005
Ipsilateral	4,2 – 5,1	1,6 – 14		Hardell et al., 2003; Hardell et al., 2005				
Contralateral	3,7 – 4,9	1,2 – 9,8		Hardell et al., 2003; Hardell et al., 2005				
Ipsi/contralateral	5,6	0,6 – 52		Hardell et al., 2003	3,9	0,8 – 19		Hardell et al., 2005
Digital technology Latency								
> 1 – 10 years Use side/VS location	2,7	1,3 – 5,7		Hardell et al., 2005	1,7	0,9 – 3,5		Hardell et al., 2005
Ipsilateral	2,9	1,4 – 6,1		Hardell et al., 2005	1,5	0,7 – 3,2		Hardell et al., 2003
Contralateral					1,6	0,7 – 3,7		Hardell et al., 2005
Ipsi/contralateral Wireless telephone	3,5	1,1 – 11		Hardell et al., 2005				
Latency > 1 – 10 years					1,3 – 1,8	0,6 – 3,6		Hardell et al., 2003 Hardell et al., 2005
Use side/VS location Ipsilateral	2,4	1,1 – 5,1		Hardell et al., 2005	1,3	0,7 – 2,7		Hardell et al., 2003
Contralateral					1,1 – 1,4	0,5 – 3,2		Hardell et al., 2003 Hardell et al., 2005
Ipsi/contralateral					2,1 – 3,2	0,7 – 13		

*Presenting minimum and maximum values present in the association value.

a: Odds ratio

b: Relative risk

c: Confidence interval

d: Not described

e: P for trend

f: the author reports that the confidence interval does not include the 1.0.

**Table 4:** Negative associations observed in the studies which analyzed risk factors for the vestibular nerve schwannoma

Risk factor	Negative association measure			
	OR^a^ or RR^b^	CI 95%^c^	P value	Reference
Family history of cancer			Hill et al., 2004	
Lung	0,5	0,1 – 1,6		
Breast	0,9	0,3 – 2,3		
Any malignant tumor in participants < 50 years	0,6	0,3 – 1,4		
Sociodemographic metrics Education			Inskip et al., 2003b	
≪ 3 years of higher education	0,6	0,2 – 1,5		
Family income Self-reported ($1000)				
< 15 – 24.9	0,1 – 0,7	0,0 – 1,9		
Census data ($1000) < 15 – 24.9	1,0	0,5 – 2,1		
Type of health care insurance Governmental	0,0 – 0,5	0,0 – 1,2		
Marital status Widower	0,9	0,3 – 2,4		
Divorced	0,0 – 1,0	0,0 – 2,1		
Separate Religion	0,0	0,0 – 1,2		
Protestant	0,8	0,4 – 1,4		
Mormon	0,9	0,2 – 3,4		
Other Christians Place of birth	0,4	0,1 – 1,4		
Neighboring states	0,6	0,3 – 1,5		
Another state in the USA	0,9	0,4 – 1,7		
Another country	0,8	0,2 – 2,7		
Laterality				
Ambidextrous	0,5	0,1 – 1,5	Inskip et al., 2003a	
Left handed and ambidextrous Dental x-ray (after 25 years)	0,9	0,5 – 1,7	Rodvall et al. 1998	
At least annual	0,7*	0,3 – 1,9		
At least every 5 years Dental filling	0,4*	0,2 – 1,0		
6 – 15	0,9*	0,4 – 2,1		
> 15 Noise	1,0*	0,4 – 3,1		
Latency period > 13 years	0,68	0,26 −1,77	Edwards et al., 2005	
Allergic disease Eczema	0,92	0,34 −2,48	Brenner et al., 2002	
Medications	0,53	0,20 −1,42		
Chemical product	0,79	0,22 −2,84		
Autoimmune disease Rheumatoid arthritis	0,28	0,07 −1,21		
Lupus	0	0 – 1,38		
Diabetes	0,76	0,33 −1,77		
Any autoimmune disease	0,61	0,31 −1,19		
Electromagnetic waves Home appliances			Kleinerman et al., 2005	
Shaver (men)	0,6	0,2 – 1,6		
Electric blanket	0,8	0,5 – 1,3		
Electric pillow	1,0	0,6 – 1,7		
Heated water mattress	0,4	0,2 – 0,8		
Stove	1,0	0,5 – 2,0		
TV set	∞	<0,001 – ∞		
Sound system	0,6 – 0,9	0,4 – 2,4		
Air humidifier	0,8	0,4 – 1,5		
Chemical substances (gene polymorphism)			Roos et al., 2003; Rajamaran et al., 2005; Roos et al., 2006.	
ALAD1 – 2	0,9	0,4 – 1,9		
GSTM1 null	0,9	0,6 – 1,6		
GSTT1 null	0,9	0,4 – 1,8		
CYP2E1 Ins96	0,4	0,1 – 1,7		
CYP1A1 462V AG or GG	0,5	0,1 – 1,4		
CYP1B1 V432L GG	1,0	0,4 – 2,2		
EPHX1 Y113H TC	0,8	0,4 – 1,3		
GSTM3 B/B Occupation	0,0	0,0 – ∞	Rajaraman et al., 2004	
Has always worked on the occupation				
Cooks and cook aides	0,7	0,3 – 1,5		
Administrator/manager	0,8	0,5 – 1,3		
Nurse, assistant and attendant in hospital	0,9	0,4 – 2,2		
Office assistant	0,9	0,5 – 1,5		
Office workers	0,9	0,4 – 1,9		
Sales people and cashiers	0,9	0,5 – 1,5		
Waiter and barman Worked for 5 years in the occupation	0,8	0,4 – 1,7		
Administrator/manager Cell phone	0,7	0,4 – 1,3		
Regular use	0,9 – 1,0	0,51 – 1,9	Christensen et al., 2004; Lönn et al., 2004; Schoemaker et al., 2005; Inskip et al., 2001	
Regular use duration				
5 – 9 years	0,86	0,39 −1,93	Christensen et al., 2004	
≫ 10 years	0,22	0,04 −1,11		
Cumulative use < 5 years	0,9	0,7 – 1,1	Schoemaker et al., 2005	
≫ 5 years	0,72 – 0,9	0,28 −1,89	Christensen et al., 2004	
Analogue technology Regular use	0,9	0,7 – 1,2	Schoemaker et al., 2005	
Time since first regular use 5 – 9 years	0,9	0,6 – 1,3		
Digital technology Latency				
> 1 year – 10 years	1,0	0,8 – 1,2	Hardell et al., 2003	
> 10 years Side of use/VS location	0,8	0,1 – 6,7	Hardell et al., 2005	
Ipsi/contralateral Wireless telephone	0,9	0,3 – 2,7	Hardell et al., 2003	
Latency > 1 – 10 years	1,0	0,9 – 1,2	Hardell et al., 2003	
> 10 years	0,3 – 0,9	0,03 – 2,3	Hardell et al., 2005; Hardell et al., 2005	

a: Odds ratio

b: Relative risk

c: Confidence interval

A family history of cancer, investigated by Hill et al.^14^, does not represent a risk factor for the development of VS, because in the study they only found non-statistically significant (NSS) positive (stomach, colon, prostate, any malignant tumor) or negative associations (lung and breast). The same can be seen in the investigation of laterality as a risk factor, in other words, for left handed individuals there was a positive association (NSS), and for ambidextrous or ambidextrous or left handed, a negative association.

It was noticed that the higher the educational level and family income, the greater the association value (Inskip et al.^12^). For the remaining sociodemographic metrics (self-reported family income, type of medical insurance, marital status, birth place and religion), positive and negative associations (NSS) were seen.

As to the exposure to dental x-ray, risk estimates were contradictory; in one study they observed a negative association^23^ and in another, an increase in risk^24^. Now, in relation to dental fillings, there were only negative associations^23^.

Exposure to noise revealed a positive association with VS. However, when the analysis considered the occupational or non-occupational exposure, non-statistically significant results were found^23^, ^24^. The authors also noticed a risk increase for tumor both for continuous noise exposure as well as impact exposure. Noise exposure duration was equal to or higher than 15 years revealed tumors. The authors found associations (NSS) or negative associations for all the genes and variants analyzed. De Roos et al.^10^ also analyzed these factors according to age and observed a positive association between the CYP2E1 Rsal gene polymorphism in individuals aged below or at 40 years (OR= 8.1; CI 95% 1.7 – 38.9). De Roos et al.^17^, in the study carried out in 2006, also carried out an analysis according to age including the variables gender and smoking habit and noticed that the NQO1 P187S CT or TT genes polymorphisms present risk for VS in male individuals (OR= 4.8; CI 95% 1.8 – 12.8). They also noticed positive associations (NSS) for three of the five genes analyzed in the study with smokers (CYP1B1 V432L GG, EPHX1 Y113H CC and NQO1 P187S CT or TT).

Rajamaran et al.^13^ studied the role of occupation in the VS etiology. For that, the participants were distributed in 121 occupational groups. For this systematic review we only listed the occupational groups made up of more than five individuals. For individuals who had always worked as gas station attendants, buyers, sales reps, teachers and instructors had positive associations with VS. Mechanics and aides, blacksmiths, recreation professionals and physical education teachers had positive associations (NSS). There was a negative association for cooks and cook aides, administrator/manager, nurse, hospital attendant and assistant, office aide, office workers, salespeople and cashiers, waiters and barmen.

Among seven studies, four described a negative association regarding the regular use of cell phones8,18,20,26. Time analysis, in years, regarding the regular use of cell phones in these studies revealed positive associations; however, not statistically significant, or negative associations in the different classes considered. Nonetheless, if the use duration is analyzed considering only the strata with less than five years and more than five years, results are contradictory. Studies by Lönn et al.18 and Christensen et al.26 revealed negative associations for periods below five years and in the study by Inskip et al.8 both negative and positive associations (NSS) were seen.

The cumulative use of cell phones was analyzed by Schoemaker et al.^22^ and Christensen et al.^26^ and the results for periods lower than or higher than five years were also contradictory, sometimes revealing a negative association, and sometimes a positive one (NSS). In 2003, Hardell et al.^25^ observed that a latency period of more than 10 years regarding the use of analogue cell phones represents a risk factor for VS. In the publication of 2005^27^, results indicated a strong association for latency periods of less than 10 years. Nonetheless, considering the period of 5 to 9 years, the study carried out by Lönn et al.^18^ revealed a positive association (NSS), and the study carried out by Schoemaker et al.^22^ revealed a negative association. For digital cell phones, there was a positive association for a latency period from five to ten years.^27^.

The analysis of the association between the side of cell phone use and VS revealed an increased risk regardless of the side of analogue cell phone use^25^, ^27^and the ipsi/contralateral use of the digital cell phone^27^. Nonetheless, in studies carried out by Inskip et al.^8^ and Muscat et al.^20^, although the cell phone technology was not considered in the analysis, there was no relationship seen between the side of cell phone use and tumor side.

Table 5 presents the cases, according to exposure duration, and the association measures of VS and cell phone. No study assessed a long exposure time or a long latency period, and they all have reduced numbers of cases in the strata that represent the greater period of exposure. The remaining variables (average of daily use in minutes, year in which the person started using the cell phone, first technology system of the cell phone used, cumulative use, and in years - the number of calls) revealed positive (NSS) or negative associations.

**Table 5:** Results and number of cases exposed in the investigations carried out in relation to the risk factor - cell phone - for vestibular nerve schwannoma

Reference	OR	CI 95%	# of exposed cases	Exposure duration
Inskip et al. 2001	1,9	0,1 – 4,2	5	Always exposed
Muscat et al., 2002*	0,5	0,2 – 1,3	7	1 – 2 years
	1,7	0,5 – 5,1	11	3 – 6 years
Hardell et al., 2003	Analogue technology			
	1,2	0,99 – 1,5	247	> 1 year
	1,2	0,96 – 1,6	160	> 5 years
	1,5	1,02 – 2,3	61	> 10 years
	Digital technology			
	1,0	0,8 – 1,2	423	> 1 year
	1,0	0,7 – 1,4	66	> 5 years
	–	–	–	> 10 years
Christensen et al., 2004*	0,86	0,4 – 1,6	23	1 – 4 years
	0,68	0,3 – 1,4	19	= 5 years
	0,86	0,3 – 1,9	17	5 – 9 years
	0,2	0,0 – 1,1	2	= 10 years
Lönn et al., 2004*	1,9	0,9 – 4,1	14	= 10 years
Schoemaker et al., 2005	0,8	0,7 – 1,0	174	1.5 – 4 years
	0,9	0,7 – 1,2	139	5 – 9 years
	1,0	0,7 – 1,5	47	= 10 years
Hardell et al., 2005	Analogue technology			
	9,9	1,4 – 69	2	> 1 a 5 years
	5,1	1,9 – 14	11	> 5 a 10 years
	2,6	0,9 – 8,0	7	> 10 years
	Digital technology			
	1,7	0,9 – 3,5	29	> 1 year
	2,7	1,3 – 5,7	23	> 5 years
	0,8	0,1 – 6,7	1	> 10 years

* Time, in years, since the first regular use

+ Latency

+ Time, in years, since the first use

Two studies analyzed wireless telephones^25^, ^27^. For latency periods > 1 year up to 10 years, there was a positive association (NSS) or negative association according to the new classification established for this systematic review. Nonetheless, when we checked the original classification of the studies for this variable, we noticed an increase in the risk for latency periods greater than five years and less than 10 years in the study of 2003 (OR= 1,3; CI 95% 1.01-1.7). In the study from 200543 this finding was not confirmed (OR= 1.4; CI 95% 0.6-3.2). For latency periods above 10 years, both studies indicated a negative association. In relation to the side of wireless telephone use and tumor location, there was a positive ipsilateral association with the VS only in the 2005 study^27^.

## DISCUSSION

VS etiology is still obscure and rare are the investigations carried out in relation to tumor risk factors. Of the 265 studies found and used for this systematic review, only 7.5% represented investigations regarding environmental, occupational or genetic risk factors for VS.

We notice that the investigation of risk factors for VS is a recent task and may be associated with the progress in diagnostic resources and consequent raise in incidence rates which provide investigation tools and called out attention to the knowledge gap regarding the etiology of this tumor. The present investigation was restricted to a systematic review; it was not possible to do a metanalysis because the variables studied in the investigations we selected were classified in different ways or in non-comparable strata.

### Association between risk factors and vestibular nerve schwannoma

Among the 20 studies analyzed we noticed the role of 15 different exposures in the development of VS. Of these, only seven can be considered as tumor risk factors, in other words, revealed at least once a statistically significant positive association (educational level, family income, occupation, hay fever and exposure to ionizing and non-ionizing radiation and high levels of sound pressure).

Inskip et al.^12^ observed that the educational level (equal to or higher than four years of higher education) and family income (equal to or higher than 25,000 dollars) represent risk factors for VS. These results are questionable and must be interpreted carefully, because individuals with higher education and better economic situation usually have more access to health information. Consequently, we can imagine a better skill to recognize signs and symptoms of diseases and a greater clarification regarding treatment and its benefits, which reflect the search for medical care. Thus, educational level can become a confounding variable and it is not a surprise that in five studies selected the results obtained have been adjusted by this variable.

The proximity between the dental arch and the skull has led some to speculate that the exposure to dental x-ray can be associated with the development of brain tumors. Preston-Martin et al.^24^ investigated this exposure and observed a positive association for an annual x-ray, before or after 25 years of age. This finding corroborates the increase in brain tumor risk, among them the VS, observed in the studies with survivors of the A-Bomb in Japan^28^ and with individuals who during childhood received ionizing radiation for the treatment of scalp disorders, tonsils and other areas of the skull and neck^29^.

Nonetheless, results from Preston-Martin et al.^24^were not confirmed by Rodvall et al.^23^, in which a negative association was observed for individuals who underwent dental x-ray up to once a year. However, results from Rodvall et al.^23^ must be interpreted carefully, because besides a small case sample (36), the authors reported that the quality of exposure data was compromised because of the bad quality of the individuals' dental records.

Epidemiological studies on ionizing radiation report this exposure as an etiological factor for tumors in adults. According to Harley^30^, the exposure to ionizing radiation frequently damages the DNA structure and this data is directly associated with the exposure dose and to the chemical structure affected. Experimental studies with mammal cells revealed that the DNA damage can occur because of the rupture of one or the entire double helix of the DNA and also by the breaking of the chemical bond between the molecules that make it.

Brenner et al.^9^ observed a positive association between hay fever, allergy and other substances (plants, dust or animals) and food allergy and VS. The authors argue that the association with hay fever can reflect a further diagnostic investigation and consequent accidental finding of a tumor because of Eustachian tube dysfunction and otitis media symptoms associated with allergic rhinitis. Nonetheless, the authors do not rule out the possibility of hay fever or even allergy - because of the overstimulation of the immune system - promoting tumor development. The association observed between allergy and other substances or food allergy and VS can be questioned, since only individual self-reports were considered in the classification of those exposed and not-exposed. There are no reports in the literature of other studies on the role of allergic disease history for VS development. However, prior studies^31^,^32^ described a risk reduction for glioma, but not for meningioma in individuals with prior history of allergic disease.

Studies by Preston-Martin et al.^24^ point to the association between VS and occupational exposure to noise in men. Thus, results from Edwards et al.^19^, assessing individuals from both genders indicated an increased risk for the tumor. These results corroborate the hypothesis that acoustic trauma, stemming from the exposure to high levels of sound pressure contribute to tumor appearance. Experimental studies with rodents have shown that impact noise causes mechanical damage to the organ of Corti and neighboring tissues, including the VIII nerve^33^, ^34^and the Schwan cells (JT Corwin, personal communication apud Edwards et al.^19^). Corwin & Cotanche^35^ and Ryals & Rubel^36^, in studies with chicken and quails, confirmed that the ear sensorial cells are destroyed and subsequently regenerated after acoustic trauma. Thus, it is plausible that the VS stems from mechanical trauma caused by exposure to high levels of sound pressure and the consequent process of cell repair - in which cell division determines DNA error replication and allows for a disorganized cell proliferation.

Preston-Martin et al.^24^ and Edwards et al.^19^ also analyzed the type of noise and noticed a risk increase both for impact noise as well as for continuous noise, and the association value was higher for the former. The authors also noticed that for periods equal to or higher than 13 years, there was a risk increase proportional to the increase in latency period. These findings can be explained, by analogy to the damage caused by noise to the hearing system, by the phenomenon described by Hammernik et al.^33^ in experimental studies involving rodents. The authors found that impact noise can instantaneously destroy 60% of the cochlea, while continuous noise would only cause such effect after long years of exposure and also by the observation that impact noise caused more damage to the nerve and adjacent tissues.

More than a billion people use cell phones all over the world and these numbers are growing rapidly^37^. In Brazil, according to the National Telecommunications Agency (ANATEL)^38^, in May of the present year mobile telephony reached the figure of 105,090,535 subscribers. Thus, there is a public health concern regarding the effects of the exposure to radiofrequency waves on a person's health. Studies on the association between cell phones and VS are controversial. Many of the studies done have methodological limitations and the results must be interpreted carefully. We noticed that the authors analyzed different characteristics of this exposure and also classified in a different way all the variables studied. This fact also made it difficult to analyze this risk factor and can be seen in the analysis of the cumulative-use-in-hours variable. All the studies that analyzed this variable^8^, ^18^, ^20^, ^22^, ^25^, ^27^ established different strata and thus, the results were controversial, that is, analyzing periods of hours of a study that would all fit a given stratum of another study, some times the variable studied revealed a negative association regarding VS, sometimes it revealed positive associations, without statistical meaning.

The analysis of these studies point to the association between the use of analogue cell phone and VS^25^, ^27^. However, the findings are contradictory, since Harrell et al.^25^ observed a risk increase for VS in analogue cell phone users for a latency period longer than 10 years. In the study carried out in 2005 results indicated a strong association regarding latency periods shorter than 10 years^27^. Nonetheless, for digital cell phones we noticed a positive association only during the latency period from five to ten years^27^. Although the association measure in both studies indicated a strong association between cell phone use and VS, results must be interpreted carefully, because confidence intervals are broad due to small sample sizes.

Radiofrequency waves emitted during the use of cell phones are absorbed by the skin and bones around the ear and may raise tissue temperature^39^, ^40^. Such phenomenon led to speculations that a thermal mechanism could trigger or accelerate the growth of subclinical brain tumors^41^, ^42^. However, according to Rothman et al.^43^ we must consider that this temperature raise is small, around 0.1° to 0.2°C, and that radiofrequency waves are rapidly damped as they pass through the tissue, so much so that less than 10% of the power emitted penetrates up to 4-6 centimeters in the skull.

A recent study on the effects of exposure to radiofrequency electromagnetic fields^44^ revealed chromosomal aberrations in fibroblasts and an increase in intracellular free radicals. These findings allow us to conclude that radiofrequency waves can activate genes which play an important role in cell division, proliferation and differentiation support the hypothesis of genetic mutations in the development of chronic diseases, such as cancer, because of cell phone use.

The analysis of the association between cell phone use and VS showed a risk increase, regardless of the side the cell phone was used - that for analogue devices^41^, ^25^, ^27^and ipsi or ipsi/contralateral side for digital cell phones^27^. Because of cell phone use near the temporal region, it is likely that this exposure characteristic could be associated with the tumor development. Nonetheless, the findings from the studies do not point only to a VS risk increase with cell phone use ipsilateral to the tumor side. Thus, it is not very likely that the absorption rate explains contralateral tumor occurrence. The findings from these studies may have been influenced by the presence of unilateral tinnitus and hearing loss - characteristic of this disease, because it can alter use pattern and consequently interfere in the questionnaire responses.

It has been observed that all the studies which consider the variables: technology, latency and cell phone use side simultaneously, show results which indicate a positive association with VS^18^, ^22^, ^25^, ^27^.

The studies which investigated landline and wireless cell phone exposure and Vs risk found a positive association according to latency period and phone use side (ipsilateral). However, these associations reveal contradictions, because while they behave like risk factors in one of the studies^27^, in another they revealed negative associations^25^. According to Hardell et al.^27^ the use of landlines and wireless telephones has not been discussed as a risk factor for brain tumors in the study carried out regarding exposure to non-ionizing radiation. However, the author's findings - statistically significant positive association with ipsilateral phone use and tumor side, can not be explained by a memory bias.

The findings from the study carried out by Rajamarm et al.^16^, in which occupation was investigated, must be interpreted carefully because of the small number of individuals in each occupational group. Moreover, one must consider that for the occupations which revealed positive association for VS (gas station attendants, buyers, sales reps, teachers and instructors) the authors do not discuss probable characteristic exposure which may be associated with tumor development. Nonetheless, we see that in all these occupations the individuals have a greater contact with the public and thus, we can suppose that these individuals may obtain further information on the signs and symptoms of diseases and, consequently, reflect on the search for diagnosis and incidental tumor finding.

Methodological limitations of the epidemiological studies which investigated risk factors associated with vestibular nerve schwannomas

Among the studies analyzed, the limitations observed for conclusive results were: reduced sample size^14^, ^16^, ^23^, greater participation rate among the cases^18^, ^19^, ^22^, use of hospital controls^8^, ^9^, ^10^, ^12^, ^13^, ^15^, ^22^ and the interviewers were not blind to the fact that the individuals were cases or controls^9^, ^19^, ^24^.

Since the VS is a relatively rare disease^1^, ^3^, ^4^ and the design of the study chosen, in all the papers analyzed, was the case-controlled - despite the recommendation of choice for this type of study for low incidence diseases45, frequent are the criticisms regarding this design because of bias susceptibility^45^, ^46^.

One of the aspects to be considered on the analysis of findings regularity is sample size^46^, ^47^. It is not always that establishing sample size is an easy task, and much less the finding of a desirable number of individuals to provide a good statistical power to the study. In three of the 20 studies selected, it was seen that the reduced sample size and what drew our attention was that in none of the studies analyzed there was a statement about sample size calculation. Thus, the findings of these investigations are questionable and do not explain the role of the risk factors studied regarding VS development.

A greater participation of individuals from the case group when compared to the control group, as observed in three other studies, can cause a study selection bias, because the population representativeness assumption to which the study is proposed does not happen^46^, ^47^.

Memory limitations, always present in any study seeking information about past events are directly related to the respondents' cognitive skills. In many situations there is the need to count on the participation of substitute respondents, which challenges the quality of the information. There are no reports in the literature pointing to a cognitive impairment in individuals with VS,48 thus, the participation of substitute respondents can be minimized, was observed in four of the selected studies8,9,12,14.

Time interval between exposure and disease diagnosis and when the interview was carried out can also impact the individual's recollection capacity. VS has variable growth rate, signs and symptoms and, thus, diagnosis can be delayed and consequently establish a longer time span between the exposure and the disease. Of all the studies analyzed, three reported that the interview with the cases was held immediately after diagnosis^14^, ^15^, ^27^.

Information regarding past exposures can also be weakened because of the emotional and social meaning of the events investigated and by the level of details required in relation to these events. This assumption was not observed in the investigations which tested the association between cell phones and VS, since details regarding technology, side of use and latency period contributed to establish this factor as a risk for developing the disease.

The differences in response obtained between the case and control groups can cause a memory bias. This can happen because the cases usually are more motivated to report possible exposures than their healthy control counterparts and also because the cases have more opportunities to think about possible causes for the disease because of the medical visits and exams they have been through. In relation to hospital-based case-control studies one could imagine that this rumination bias was minimized because the controls were admitted to the same hospitals as the cases with a variety of non-malignant conditions. Nonetheless, this apparent advantage can also be a limitation^46^, ^47^, ^49^, since controls have varied diagnoses and use different drugs which can also somehow be associated with the disease under investigation. For instance, Brenner et al.^9^, in their study about the past of allergy and autoimmune disease and VS, did not investigate the use of medication and, thus, the authors did not rule out their interference in the results attained. The care in excluding controls with diseases that could be associated with the study hypothesis was taken in one of the investigations selected^16^.

Case-control studies are recommended in incidental cases in order to mitigate a possible survival bias. Most of the studies analyzed (18) included only incidental cases and in two of them there were no reports whether the cases were incidental or prevalent. VS is a benign tumor with a low lethality rate^1^, ^2^, ^4^ thus, there is no risk of losing sample individuals during the study, making it possible to include prevalent cases as well.

Interviewers' knowledge judgment and values may also interfere on the responses of the individuals and distort the results. This concern was visible in most of the studies analyzed since the interviewers were trained and blinded in relation to the situation of the interviewee - as for being a case or a control. Such measures aim at minimizing the effect of a possible checking bias. In the studies led by Brenner et al.9, Preston-Martin et al.^24^and Edwards et al.^19^ it was not possible to blind the interviewers regarding interviewee status. However, in the first they were not informed about study hypothesis and in the other ones the interviewers were properly trained.

### Problems measuring exposure to the investigated risk factors

Checking past facts in order to estimate exposure to a given risk factor is not only impacted by memory^45^but it is also directly associated to the instrument used and the collection technique. Most of the studies selected for this systematic review (14) used only questionnaires (instruments) in order to check exposure and it was only in five of them that the information collected was validated using other data sources. In 17 studies there were personal interviews, which allows for a certain interaction between researcher and interviewer, and it also helps explain possible doubts which could crop up because of the very difficulty of understanding by the interviewee. Another advantage of this technique is the employment of questionnaires by the researcher in such as way as to guarantee that they will all be entirely filled out, minimizing the exclusion of individuals due to incomplete data.

Of the two studies which investigated the risk factor of dental x-rays for VS, both used questionnaires in order to obtain information regarding the number of radiographs performed and, in one of them, these data were validated by the individuals' dental records. Nonetheless, none of the studies reported data regarding the dose of radiation to which the individual was exposed.

The validation of the information provided by the individual was carried out in only one of the studies which investigated the risk of exposure to high levels of sound pressure to VS development - in this study they considered only those exposed individuals who reported occupations listed by the national research on occupation risk carried out in the USA (NOHS) as those which involved exposures to high levels of sound pressure. In the study in which a family history of cancer was investigated, the authors confirmed the cancer cases reported by the individuals.

Regarding measuring exposure to high levels of sound pressure we must also consider the fact that workers exposed to noise can develop hearing loss and, therefore, normally undergo periodic tests more often than the population in general. This fact can facilitate diagnosis and increase tumor incidence rate in this specific population. Moreover, there are possibilities for these individuals to establish a relationship between the exposure and the disease and thus determine greater response accuracy. In estimating noise exposure, the investigation of potential effect confounding or modifying factors must also be considered, because in many occupations there is concurrent exposure to chemical products. This fact was seen in only one of the studies analyzed which investigated noise exposure as a risk factor.

The results attained in the studies which investigated the role of allergic disease and autoimmune history must be interpreted carefully, because the authors did not check the exposure to drugs used by the individuals which may act as confounding of modifying factor to the association effects. We must stress that for only one of the investigated allergic disorders the medical diagnosis was considered in order to classify exposed individuals.

Investigations about cell phone and wireless phones also used only questionnaires in order to check exposure. Result divergences and evidence contradictions in these studies may be associated to the recent introduction of cell phones in the market, in other words, the temporal relationship between exposure measurement and disease can not contemplate the latency period necessary for tumor development. Moreover, it is likely that the small number of individuals in the strata with greater time of exposure may also have contributed to result scatter and lack of uniformity. Such particularity was also noticed in the study which investigated the association between occupation and VS, because the authors created 121 occupational groups in order to classify exposure and in many of them there was a reduced number of participants.

Another aspect to be analyzed is the cell phone technology used by the individual. In the first years, after mobile phones were introduced in the market, only analogue technology devices were available, and today there are also digital cell phones available. The frequency ranges used by analogue and digital cell phones are different; therefore it is desirable to have accurate information regarding the use of one or the other technology. Nonetheless, the investigation of such exposure may not be accurate because of our lack of knowledge regarding the technology used by the individual and also by the difficulty in remembering for how long each one of the technologies was used. We also have to consider the use of both, as well as the time during which cell phone use was discontinued. Thus, it is likely that a greater accuracy regarding cell phone exposure can only be reached through the validation of information provided by the individuals with data from cell phone carriers. Considering the fact that VS is usually unilateral, when considering cell phone exposure one has to consider the side on which the cell phone is used. Nonetheless, this exposure is not always accurately measured, since hearing loss or tinnitus may change cell phone use patterns.

The exposure to electromagnetic waves from household appliances was also investigated in the questionnaire which included the frequency and duration of use of the different devices. Notwithstanding, in establishing such time, use discontinuation was not considered, since such fact was not investigated.

Digital-manual laterality investigation as a risk factor for VS was carried out by means of two simple questions presented personally to the participants at the time of hospital admission. In such exposure testing, one must consider the impact of sociocultural factors used to establish or to modify the digital-manual laterality.

Regarding exposure checking to different sociodemographic indicators, besides the answers given by the individuals, census data regarding family income were also considered in the classification of those exposed and not exposed. For the remaining factors investigated, only the interviewees' reports were considered for exposure classification.

## CONCLUSIONS

Education (equal to or higher than four years of higher education) and family income (equal to or higher than 25,000 dollars), occupation, hay fever and exposure to ionizing and non-ionizing radiation and high levels of sound pressure are risk factors for VS, according to the association values presented in the studies we analyzed here. Nonetheless, the analysis of the methodological quality and findings accuracy through confidence interval and biologic likelihood between exposure and outcome, suggests the exposure to ionizing (dental x-ray) and non-ionizing (cell phone) radiation and high levels of sound pressure as more important risk factors. Although the studies which reveal these factors as risks for VS development were better carried out from the methodological standpoint and their findings were more accurate, one must assess tumor development latency period, the reduced number of exposed individuals and the problems encountered with exposure checking are still not clear about the role of these factors in the disease etiology. Thus, future investigations are necessary in order to have a better understanding of such issue, which findings can help establish preventive measures, as well as contribute to an earlier diagnosis, before signs and symptoms ensue and, consequently, impact on the reduction of sequelae stemming from surgical intervention.
